# Experimental Horizontal Transmission of *Enterospora nucleophila* (Microsporea: Enterocytozoonidae) in Gilthead Sea Bream (*Sparus aurata*)

**DOI:** 10.3390/ani11020362

**Published:** 2021-02-01

**Authors:** Amparo Picard-Sánchez, M. Carla Piazzon, Itziar Estensoro, Raquel Del Pozo, Nahla Hossameldin Ahmed, Oswaldo Palenzuela, Ariadna Sitjà-Bobadilla

**Affiliations:** 1 Fish Pathology Group, Instituto de Acuicultura Torre de la Sal (IATS-CSIC), 12595 Ribera de Cabanes, Spain; amparo.picard@csic.es (A.P.-S.); carla.piazzon@csic.es (M.C.P.); itziar.estensoro@csic.es (I.E.); raquel@iats.csic.es (R.D.P.); oswaldo.palenzuela@csic.es (O.P.); 2 National Institute of Oceanography and Fisheries, NIOF, Cairo 11562, Egypt; dr_nahla83@hotmail.com

**Keywords:** *Sparus aurata*, experimental transmission, *Enterospora nucleophila*, Teleostei, microsporidia, aquaculture, temperature, immunosuppression, histopathology

## Abstract

**Simple Summary:**

*Enterospora nucleophila* is a microsporidian parasite infecting gilthead sea bream (*Sparus aurata*) that has been associated with increased mortality, growth arrestment and emaciation in aquaculture settings. The investigations on this parasite depend on material from spontaneous outbreaks in fish farms, which hampers advancements in infection research and control. In the present study, different ways of horizontal transmission were performed in order to establish an in vivo transmission model. Parasite transmission was achieved with all the assayed routes, but with milder clinical signs than in natural infections.

**Abstract:**

*Enterospora nucleophila* is a microsporidian enteroparasite that infects mainly the intestine of gilthead sea bream (*Sparus aurata*), leading to an emaciative syndrome. Thus far, the only available information about this infection comes from natural outbreaks in farmed fish. The aim of the present study was to determine whether *E*. *nucleophila* could be transmitted horizontally using naturally infected fish as donors, and to establish an experimental in vivo procedure to study this host–parasite model without depending on natural infections. Naïve fish were exposed to the infection by cohabitation, effluent, or intubated either orally or anally with intestinal scrapings of donor fish in four different trials. We succeeded in detecting parasite in naïve fish in all the challenges, but the infection level and the disease signs were always milder than in donor fish. The parasite was found in peripheral blood of naïve fish at 4 weeks post-challenge (wpc) in oral and effluent routes, and up to 12 wpc in the anal transmission trial. Molecular diagnosis detected *E. nucleophila* in other organs besides intestine, such as gills, liver, stomach or heart, although the intensity was not as high as in the target tissue. The infection tended to disappear through time in all the challenge routes assayed, except in the anal infection route.

## 1. Introduction

Microsporidia are obligate intracellular (intracytoplasmic and/or intranuclear) eukaryotic, spore-forming parasites that can infect almost all types of animals, from vertebrates (including humans) to invertebrates [1]. The impact of these parasites in aquaculture, fisheries and fish research facilities has been widely documented [1,2,3]. Despite being extraordinarily widespread and with significant impact on human and livestock health, microsporidian infections are poorly understood.

Microsporidian life cycles consist of two general developmental phases: merogony and sporogony. Meronts multiply inside the infected host cell, eventually forming sporonts and spores, which are ultimately released from the host and transmit the infection. The spore is both the infectious and the resistant stage thanks to its thick chitinous layer [4,5]. It is extremely resistant to environmental stress and lysis, allowing the organism to maintain viability in the aquatic environment for extended periods [6,7,8] and to resist digestive enzymes in the gastrointestinal tract of the hosts. In general, fish microsporidia are transmitted directly, presumably *per os*, by ingestion of infected tissues or spores present in the water, or through the penetration of the skin [9]. Different routes of horizontal transmission have been tested for microsporidia in fish, such as oral intubation [10,11,12], cohabitation [11,12], effluent transmission/bath with parasite stages [8,13,14,15], and intracoelomic or intramuscular injections [10,12,16]. Several studies have achieved direct horizontal transmission, such as with *Enterocytozoon salmonis* [17], *Loma salmonae* [12], *Glugea anomala* [18], *Glugea plecoglossi* [19] or *Glugea stephani* [10,20]. However, for other microsporidian species, an intermediate host is needed to complete their life cycle [21,22].

*Enterospora nucleophila* is an enteric microsporidian that affects mainly the intestine of gilthead sea bream, provoking anorexia, cachexia, and even death [23]. Recently, a negative correlation between the intensity of *E. nucleophila* infections and the condition factor (CF) of infected fish has been described [24], which demonstrates the growth arrestment induced by the parasite. The microsporidium is located mainly in the epithelial layer of intestinal mucosa and in the submucosa, infecting the cytoplasm of enterocytes and macrophages and the nucleus of enterocytes and rodlet cells, although other organs can be infected [23,24]. The difficulty of diagnosing this disease, due to the very minute spore size and its intranuclear location, has recently been overcome by the development of qPCR and ISH diagnostic methods, which allow one to detect very low infection levels that can be considered negative by histology [24,25]. Another impediment to study this emerging disease is the difficulty to isolate the minute spores and to obtain an in vitro culture. Therefore, the aim of the current work was to develop an in vivo model of transmission that could provide a source of parasite, to test treatments and prophylactic approaches and to study the host–parasite interactions in depth under controlled conditions.

## 2. Material and Methods

### 2.1. Donors and Naïve Recipient Fish

Three different lots of gilthead sea bream (GSB) naturally infected with *E. nucleophila* (*n* = 300 for Lot 1 and *n* = 200 for Lots 2 and 3) were used as donors (D) (Table S1). They were harvested from commercial sea cages in the western Mediterranean Sea and displayed the typical growth arrestment and emaciation signs associated with this microsporidiosis [23]. The smaller, wasted fish were handpicked and transported alive to the Institute of Aquaculture Torre de la Sal (IATS-CSIC, Castellón, Spain) facilities. Upon arrival, a subsample was sacrificed by overexposure to the anaesthetic (MS-222, 0.1g/L; Sigma), and tested for the presence of *E. nucleophila* in the intestine by qPCR or by histology (see more details below). Donors for each trial were selected from these infected fish stocks according to the clinical signs described previously: cachexia, swollen abdominal cavity, and thinned intestinal walls in the necropsy [23,24], and these fish were generally positive for *E. nucleophila* by qPCR and histology.

Healthy GSB juveniles from a commercial nursery were used as recipient fish (R), and kept in the same facilities in 5 μm filtered and UV-treated sea water (salinity 37.5 g/L) under natural temperature and photoperiod at our latitude (40°5′ N; 0°10′ E). The absence of *E. nucleophila* in R stocks was confirmed by qPCR according to Picard-Sánchez et al. [24]. Fish were fed ad libitum a commercial diet (BioMar, Palencia, Spain) and they were kept according to the Guidelines of the European Union Council (Directive 2010/63/EU), the Spanish RD 53/2013, and the CSIC National Committee on Bioethics under approval number 2018/VSC/PEA/0240.

### 2.2. Samplings and Parasite Diagnosis

Before all samplings, fish were starved for two days. Weight and length were registered in all samplings and the condition factor (CF = (100 × body weight)/length^3^) was calculated. The prevalence of infection was calculated as the percentage of *E. nucleophila*-positive fish out of the total of sampled fish in each experimental group.

Parasite diagnosis was carried out as previously described [23,24,25]. Briefly, for histology processing, pieces of intestine were fixed in 10% buffered formalin and embedded in paraffin or in methacrylate resin (Technovit 7100, Kultzer, Wehrheim, Germany). Tissue sections (4–5 μm for paraffin and 1 μm for resin samples) were stained with Giemsa or with 0.1% calcofluor white M2R stain (CW) and 0.1% Evans blue [25]. Slides were dehydrated, mounted in DPX, and examined using bright-field and fluorescent microscopy under UV excitation light, respectively. In histological slides, parasite infection was evaluated for presence or absence of sporogonial and merogonial stages. Intranuclear infections, typically affecting enterocytes and rodlet cells (RCs), were discriminated from cytoplasmic infections affecting enterocytes and phagocytes at the epithelium and lamina propria submucosa [23,25]. Observations were made up to 1250× magnification.

Molecular diagnosis was performed by qPCR as previously described [24]. Briefly, non-lethal samples (NL-qPCR) consisted of a sample of rectal mucosa taken from anaesthetised with a cotton swab, and/or 3–4 µL blood samples drawn using 0.5 M EDTA-coated syringes. Both types of NL-qPCR samples were deposited in 200 µL of lysis buffer and conserved at 4 °C. Lethal samples (L-qPCR) consisted of small pieces of intestine tissue (50–100 mg) in 200 µL of lysis buffer, or an aliquot (100–200 µL) of tissue homogenate in Tris-EDTA buffer (10 mM Tris, 1 mM EDTA, pH 8.0), obtained with a laboratory blender. DNA was extracted from the samples with a robotic system (EpMotion 5070, Eppendorf, Hamburg, Germany), using DNA extraction kits for tissues or blood (Nucleospin, Macherey-Nagel, Germany). Quantification and purity of DNA samples were assessed with a spectrophotometer (Nanodrop 2000c, Thermo Scientific, Spain). *E. nucleophila* detection in these samples was carried out with specific primers for *E. nucleophila* SSU rRNA gene [24], and the intensity of infection was determined using the reactions Ct values, with a positive cut-off value of Ct = 38. Standard curves with known numbers of the target gene were included in each plate and used as intra and inter-assay quality controls.

### 2.3. Experimental Transmission Trials

Four different experimental transmission trials were carried out, using donors from three different outbreaks at commercial sea cages, obtained in February 2013, March 2014 and April 2017 (Table S1). The experiments within each trial focused on specific routes or conditions for the transmission and/or the downstream diagnostic tests applied. Details of these trials are summarised in Table 1.

Transmission by water effluent exposure (EF) was performed by connecting tanks holding naïve recipient (R) fish to receive the effluent water from another tank containing donor (D) fish, until the end of the experiment. The D tank was the only tank that received inlet water. Transmission by cohabitation (CH) was performed holding in the same tank R and D fish tagged with passive integrated transponders (PIT-tags). Transmission by oral (O) or anal (A) intubation was made as described in Estensoro et al. [26], with minor modifications. Briefly, intestinal scrapings of D fish were diluted with sterile Hank’s balanced salt solution (HBSS), and intubated orally or anally to naïve R fish, using a blunt and narrow cannula. Water flow was adjusted to three renovations per day in all the trials.

*Trial 1:* In this first small-scale trial, two routes were assayed: oral (O-1) and cohabitation (CH-1) using 50 g fish (initial body weight) (Table 1). In O-1, fish were inoculated with 0.6 mL/fish. Samplings were carried out between 8- and 20-weeks post-challenge (wpc). In this trial, only histological diagnosis was performed.

*Trial 2*: Two routes were assayed: oral (O-2) and effluent (EF-1, EF-2) using 40 g (O-2 and EF-1) and 80 g (EF-2) fish (Table 1). The oral inoculum volume used was 1 mL/fish. Samplings were carried out between 4 and 10 wpc. In O-2 and EF-1, different diagnostic methods were used and compared: histology (CW-paraffin sections, only in the first sampling) and molecular diagnosis. In the first sampling, molecular diagnosis was carried out by NL-qPCR from blood and rectal swabs. In the second sampling, L-qPCR was performed from blood and intestinal samples, either from separate intestinal segments (anterior (AI), middle (MI) and posterior (PI)) or from the whole intestine homogenate to compare the results. In EF-2, the diagnosis was performed by NL-qPCR of rectal swabs in the first sampling, and by L-qPCR of whole intestine in the second sampling.

*Trial 3:* In this trial, the oral route was tested in fish of 10 g of initial body weight. Here, a group of immunosuppressed fish (O-3-I) was included and compared to non-immunosuppressed fish (O-3) (Table 1). Immunosuppression of gilthead sea bream was induced with a synthetic corticosteroid, triamcinolone acetonide (TA) (Sigma) [27]. Briefly, 24 h before the challenge, O-3-I fish were intracoelomically injected with 100 μg/g body weight of TA suspension in 0.85% saline. Non-immunosuppressed fish received 0.85% saline. The oral inoculum volume was 0.25 mL/fish (26.3 μL/g of R fish body weight). Diagnosis was performed by NL-qPCR of intestinal swabs at 4 wpc, and by L-qPCR of the whole intestine at 12 wpc.

*Trial 4:* In this trial, the anal intubation route was tested (A-1) (Table 1) using an inoculum volume of 1 mL/fish in 30 g fish. Three sampling points were set at 4, 8, and 12 wpc. Diagnosis was performed by conventional histology and CW staining (paraffin sections) from the three different intestinal segments and by L-qPCR from the posterior intestine, aiming to compare the three diagnostic methods. In addition, L-qPCR with samples from other tissues (stomach, blood, heart, brain, spleen, gall bladder, head kidney, posterior kidney, liver, gills, and swim bladder) was also performed to have an overview of the systemic expansion of the parasite.

### 2.4. Statistics

Statistical analyses were performed with GraphPad Prism 8 (GraphPad Software, Inc., San Diego, CA). Differences in the prevalence of infection were calculated with Chi-squared test, and one-way ANOVA or Student’s t test for pairwise comparisons was used to compare biometric parameters among the groups of each trial and sampling. In all cases, *p* < 0.05 was considered statistically significant.

## 3. Results

### 3.1. Trial 1

**Figure 1:**
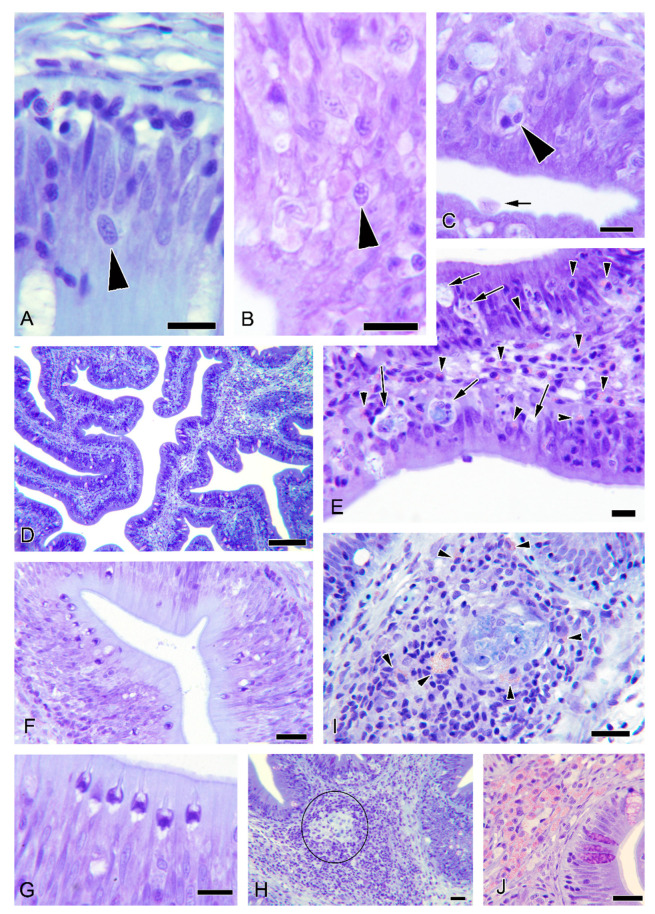
Photomicrographs from histological sections of resin-embedded gilthead sea bream intestines, experimentally exposed to *Enterospora nucleophila* by oral intubation and cohabitation challenges: O-1 (**A**–**C**,**F**,**G**) and CH-1 (**D**,**E**,**H**–**J**). Minute merogonial stages in the host-cell nuclei (**A**,**B**) or cytoplasm (**C**) (arrowheads). Spores are visible within a cell in the lumen (arrow in (**C**)). Panoramic view showing conspicuous hypercellularity of the epithelium and lamina propria-submucosa (**D**). Vacuolisation and pyknotic nuclei of enterocytes (arrows), intraepithelial lymphocytes, and infiltration of eosinophilic granular cells (arrowheads) (**E**). Proliferation of rodlet cells (**F**,**G**). Formation of macrophage aggregates (**H**,**I**) with lymphocytic infiltration (circle in (**H**)) and eosinophilic granular cells (arrowheads in (**I**)). Severe infiltration of eosinophilic granular cells in the lamina propria-submucosa (**J**). Anterior intestine (**A**–**C**,**I**,**J**); middle intestine (**D**,**E**,**H**); posterior intestine (**F**,**G**). Scale bars = 10 µm (**A**–**C**,**E**,**G**); 20 µm (**F**,**I**,**H**,**J**); 100 µm (**D**). Staining = Giemsa.

In O-1, the prevalence of infection (by histology) was 14.3% (2/7) at 8 wpc, but it dropped to 0% at 20 wpc. Most of the detected parasite stages were early merogonial stages, located in intranuclear position at the intestinal epithelium (Figure 1A,B). Immature spores were rarely found in the cytoplasm of infected cells and were released to the gut lumen in one specimen (Figure 1C). *E. nucleophila* in CH-1 was not detectable by histology until the last sampling (20 wpc) with a prevalence of 8.3% (1/12).

### 3.2. Trial 2

In this trial, we compared different diagnostic techniques in oral (O-2) and effluent (EF-1) transmission challenges, as well as checked the effect of fish size in EF-2. The results are summarised in Table 2.

In O-2 and EF-1, histological diagnosis by CW-paraffin sections at 4 wpc was negative (not shown). However, NL-qPCR of intestinal swabs resulted in 70% and 66.7% of positive fish in O-2 and EF-1, respectively. Molecular diagnosis in blood showed 93.3% prevalence in EF-1 at 4 wpc, but in O-2, no infection was found in blood until the second sampling. Prevalence and intensity (median Ct) values were always higher in EF-1 than in O-2. Molecular diagnosis of different intestinal segments and whole intestine was performed in the second sampling. The lowest Ct values achieved in O2 were 34.4 at the AI, and in EF-1 31.9 at the AI and 30.9 at the MI. The median Ct of the different segments varied very slightly between 36 and 36.4 in O-2, and in EF-2, variation was higher, between 33.1 and 36.2. Prevalence at AI, MI and PI was 50%–30%–60% in O-2 and 60%–50%–50% in EF-1, whereas that of the whole intestine was 60% and 100%, respectively. These results show that, as expected, qPCR of whole intestine always yields the highest prevalence values.

In EF-2, prevalence of infection by NL-qPCR reached 80.6% at 4 wpc, but it dropped to 11.7% in the second sampling. Regarding clinical signs, differences in biometric parameters were detected in both sampling points, showing that R fish significantly decreased in size, length and condition factor when compared to the non-challenged control group.

### 3.3. Trial 3

In this trial, we assayed the oral intubation route (O-3) and the effect of immunosuppression (O-3-I) in an attempt to increase the susceptibility of the fish and achieve severe infections. The results of this trial are summarised in Table 3.

Few animals were found infected by L-qPCR of intestinal samples. The prevalence of infection in the immunosuppressed group was 10% at 4 wpc and 0% at 8 wpc. In the non-immunosuppressed group, prevalence was 0% and 5.5%, at 4 and 8 wpc, respectively. Biometric values (weight, length and CF) were significantly lower in both R groups, particularly at 8 wpc, when compared to the control non-challenged groups.

Considerable mortality was recorded during this trial, reaching 85% and 40% in R immunosuppressed (O-3-I) and untreated (O-3) groups, respectively. Unfortunately, samples from carcasses could not be recovered for qPCR testing, and the infectious status of the dead fish was unknown. This notwithstanding, the mortality registered in non-challenged groups was 20% in the immunosuppressed control group and 9.1% in the untreated control group. In R fish, mortality and immunosuppression were significantly associated (Chi-squared test, *p* < 0.0001). This association was not found in the non-challenged groups (Chi-squared test, *p* > 0.05).

### 3.4. Trial 4

**Figure 2:**
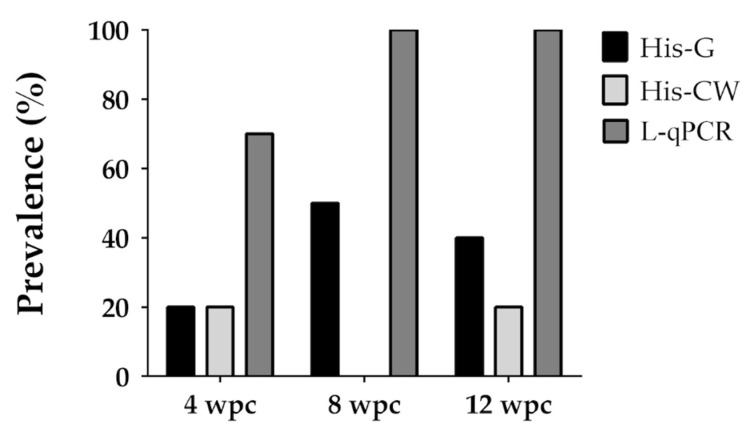
Prevalence of infection of gilthead sea bream experimentally challenged with *Enterospora nucleophila* by anal intubation (A-1, Trial 4) and sampled at different weeks post-challenge (wpc). Parasite detection in the intestine was carried out from lethal samples by histology with Giemsa (His-G), Calcofluor White (His-CW) staining, and molecular diagnosis (L-qPCR). Fish with Ct values below 38 were considered qPCR-positive.

In this trial, the anal intubation route was explored, and the infectious status was monitored by qPCR in different organs, in monthly intervals along a 3-month period after the challenge. A comparison between histological and molecular diagnosis was also performed in intestinal samples. The results of the parasitological diagnosis are summarised in Table 4 and Figure 2. No statistically significant differences between the challenged and non-challenged control groups were detected for biometrical parameters at any sampling point (data not shown). No mortality was registered.

The tissues with higher prevalence of infection by L-qPCR were the stomach and the intestine, reaching 80–100%. The prevalence of infection increased in the intestine and remained high with time, whereas in the stomach the prevalence was higher in the first sampling, dropping to 20% in the last sampling. In lymphohematopoietic organs, the highest prevalence of infection was 20–30% in spleen and kidney, respectively, at 4 wpc, but these values dropped to 10% subsequently in both tissues. Gills and liver also reached high values (up to 80%) at certain samplings, but a gradual increase or decrease with time was not detected. Regarding the intensity of infection, the lowest Ct values (highest intensity of infection) were consistently observed in the intestine (Table 4).

Detection of the parasite by histology was less sensitive than the qPCR (Figure 2). In the intestine, spores were detected in CW-paraffin sections in 20% of the samples at 4 and 12 wpc, but all samples were negative in the intermediate sampling. In Giemsa-stained sections, merogonial and sporogonial stages of *E. nucleophila* were found with a prevalence of 20%, 50% and 40% at 4, 8, and 12 wpc, respectively. In most cases, parasite stages were scarce. Most fish, although with no detectable *E. nucleophila* stages, displayed common histopathological conditions in variable degrees: cells with cytoplasmic debris, abnormalities in the nucleus of intestinal epithelial cells, hypercellularity in epithelium or lamina propria-submucosa layer, vacuolated cells and increased number of eosinophils.

For a quick comparison of all trials and challenge routes, a summary of the prevalence of infection is presented in the last column of Table 1. A common pattern found in most of the exposed fish (R) was a higher prevalence of infection at shorter times post-challenge than at longer exposure times. However, in EF-1 and A-1, prevalence increased over time.

### 3.5. Histopathological Observations

Common histopathological features were observed in R fish with no detectable *E. nucleophila* parasitic stages from trials 1 and 4: (1) conspicuous hypercellularity in the intestinal epithelial layer (Figure 1D) with disorganization of the palisade structure, in which enterocyte nuclei presented anomalous chromatin patterns and nuclear sizes, including both anisokaryosis and macrokaryosis; (2) presence of epithelial vacuolated cells with pyknotic nucleus and cytoplasmic debris (Figure 1E); (3) proliferation of rodlet cells in the epithelium (Figure 1F,H); (4) hyperplasia of the lamina propria-submucosa with lymphocytic infiltration, sometimes surrounding macrophage aggregates (Figure 1I,H); (5) proliferation of eosinophilic granular cells in the lamina propria-submucosa with certain infiltration in the mucosa (Figure 1J).

### 3.6. Additional Observations in Donor Fish

During the different trials, the lots of fish used as donors (D) were periodically sampled, and their infection status evaluated by histology and/or qPCR. Results of those samplings are summarised in Table S1. In general, a progressive loss of infection was registered throughout time in these lots, and variations in the diagnostic sensitivity among techniques were observed. Molecular detection was the most sensitive technique, followed by the examination of Giemsa-stained resin slides, and examination of CW-paraffin sections. However, differences in the clearing of the infection with time were observed depending on the D lot. All the D fish from lot 1 remained positive by qPCR even after 386 days, whereas, in the remaining trials, the prevalence of D fish dropped from 100% to values as low as 18.2% at subsequent samplings.

## 4. Discussion

Microsporidian parasites are widespread in the animal kingdom, infecting mainly arthropods and fish [28]. Their importance in cultured fish has increased in parallel with the worldwide growth of aquaculture production [2]. In cultured gilthead sea bream, several microsporidian infections have been described [29,30,31], being *E. nucleophila* the most recent and an emerging problem in the industry [23]. Information about natural *E. nucleophila* transmission was scarce when the current trials started, however, there was a clear indication of the existence of long pre-patent period (several months) in which fish harbouring the parasite do not show any disease signs. Indeed, farmed fish when first diagnosed by qPCR are asymptomatic, and then three months later showed the typical infection signs (pale organs, arrested growth, cachexia) and severe histopathological damage [24].

In this work, we succeeded in transmitting the parasite by oral, effluent, cohabitation and anal challenge, as the parasite could be detected in R fish by NL- and L-qPCR of intestine, though, in many cases, it was not detected by conventional histopathological examination. We did not test intracoelomic and intramuscular injections, despite being successful in other fish models [12,16]. The prevalence of infection achieved varied highly depending on the transmission method and the infection status of the donor (D) fish. As a general trend, higher prevalence was obtained at shorter times by oral and anal intubation routes, and longer times were needed to detect the parasite with the effluent route. Differences in the transmission rate between anal and oral routes may be due to the direct inoculation into the target organ. Indeed, anal intubation already has been described as the quickest route of transmission for another enteric parasite of gilthead sea bream, *Enteromyxum leei* [26]. In most trials, a decreasing trend of detectable parasite levels was observed, which paralleled the findings observed in most donors at longer times (Table S1), as well as the long-term epidemiological studies performed by the authors in fish farms (unpublished data). This could be explained in part by the temperature increase along the trials, which would allow the fish to activate a successful immune response and clear off the parasite. Signs of infection and prevalence in the field tend to disappear between April and November, while the current challenges ended in late spring or summer (see temperature ranges in Table 1). In fact, the common observations in R fish of intestinal epithelial cells with debris, hyperplasic lamina propria-submucosa with leucocyte infiltration, and the development of macrophage aggregates in the lamina propria-submucosa, could be interpreted as signs of a strong host reaction in agreement with this hypothesis. Generally, low water temperatures are described to inhibit microsporidian development in fish [2], but in warmer latitudes the parasites must reach a compromise between the immunological status of the host and optimal parasite reproductive temperature. This is the case of *Pleistophora aegyptiaca,* which infects greater lizardfish (*Saurida tumbil*) in the Red Sea [32], reaching seasonal infection peaks in winter. In line with this hypothesis, we have recently described that the adaptive immune response of gilthead sea bream decreases with temperature [33]. Therefore, further studies are needed using constant low water temperature typical of winter months.

The fact that fish with no apparent clinical signs (both D and R) have detectable levels of the parasite by qPCR for long periods has epidemiological implications in aquaculture, as they may behave as asymptomatic carriers of the parasite. Specific studies using ISH methods for *E. nucleophila* have also suggested the risk of incomplete infection clearing and reactivation of covert infections with this parasite [25].

In nature, microsporidian infections may range from asymptomatic to severe, even resulting in death [2,19,34]. However, these parasites are generally considered as having a relatively balanced relationship with their hosts, normally causing chronic subclinical infections, but presenting large oportunistic potential [35,36]. Indeed, immunodeficient mammals are classic examples in which microsporidian infections can result in serious clinical diseases. This highlights the important role of immune competence in alleviating health effects of microsporidiosis at individual- and population-level [37]. Outbreaks by *E. nucleophila* in gilthead sea bream farms coincide, in most cases, with winter temperatures, when the performance of the fish immune system is lowered [38]. Immune status of fish is highly affected also by cortisol levels, which can be naturally raised by handling, overcrowding, water temperature changes, etc., or induced by injection of analogues such as triamcinolone acetonide (TA). In this study, we tested an artificial immunosuppressor (trial 3), resulting in higher prevalence of *E. nucleophila* at short-time sampling points, although low overall prevalence of infection was achieved in this trial, probably due to the use of D fish with long-lasting infections in recovery stages. Further experiments using overtly infected D fish would probably improve the results. Other studies have described the rise in pathogenicity for fish microsporidians after triggering events. For example, in *P. neurophilia* zebrafish infections, clinical signs and mortality usually become apparent after stressful operations such as crowding or shipping [39], or after experimental immunosuppression by irradiation [40]. A different study revealed that intensity of infection by *Loma salmonae* can increase after a dexamethasone treatment in rainbow trout [41].

Along the different trials, different diagnostic methods were used, and it turned out, as expected, that qPCR was the most sensitive. As an example, in trial 1, prevalence determined by histology was about 1/3 of the value obtained by qPCR in similar subsequent challenges (trial 2). When parallel samples were analysed by histology and qPCR from R fish (Figure 2) or from D fish (Table S1), the prevalence values were always lower for histological methods. Indeed, when histopathological examination of the R fish from other trials was conducted, most samples did not show parasite stages, but only the histopathological alterations typical of the infection. Only in the anal challenge (trial 4), when prevalence by qPCR reached 100%, unambiguous merogonial stages were detectable in the intestine of 40% of the R fish and spores in 20% at 12 wpc.

In trial 2 (O-2 and EF-1), L-qPCR samples were taken from three intestinal segments as well as from whole intestine. The differences in the prevalence and intensity of infection confirmed what was already detected in naturally infected fish [24], that the highest intensity of infection was detected in the anterior and middle segments, but individual patterns varied widely, and only a whole-intestine homogenate could reveal the true prevalence of the stock (100% at S2 of EF-1, while it was 60% in AI, and 50% in MI and PI).

Considering the results obtained by qPCR in blood samples, it is tempting to suggest that blood could represent a systemic dispersion route within the host. In fact, in EF-1, the parasite was detected in blood with very high prevalence (93.3%) as early as 4 wpc, which decreased considerably at 10 wpc. Similar prevalence values were found at 8 wpc in blood during O-2 challenge and the lowest prevalence detected in blood was in the anal challenge (20% at 8 wpc). These observations are consistent with the detection of *E. nucleophila* spores in peripheral blood reported in a previous study [25]. Systemic transportation of parasites via peripheral blood has also been reported in other fish microsporidians. Studies on xenoma-forming microsporidians such as *Glugea* spp. and *Loma* spp. have shown that host cells within the blood lineage (e.g., macrophages) provide the transportation to the gills, where the final development occurs [42]. In *E. nucleophila* infections, macrophages are indeed commonly infected, mainly present in the intestinal lamina propria-submucosa but also in blood vessels [23,24]. These cells are involved in the parasite clearing mechanisms through the development of MMC in the intestine submucosae, where parasite debris accumulates in advanced infections [25]. However, they may also play a role in the dispersion and reinfection within the host. Other blood cells, such as erythrocytes, which often exhibit anomalous chromatin patterns in infected fish, could also participate in this systemic dispersal.

The disease signs of this microsporidiosis in naturally infected fish consist of arrested growth, poor condition and emaciative syndrome during farm outbreaks, in association with increasing mortalities. These signs were clearly observed in donor fish brought to our facilities, reaching mortalities higher than 50% in most cases and showing severe growth arrestment. Interestingly, a decrease in condition factor (CF) was also observed in trials 2 and 3, as soon as 4 wpc, and in coincidence with a high prevalence of infection in R fish in the EF-2 challenge. This arrested growth still persisted one month later at the next sampling, despite the much lower infection levels found. In both O-3-I and O-3 challenges of trial 3, although very low infection levels were reached in R fish, CF was also significantly lower than in control fish. Further studies are needed to understand the mechanisms involved in this growth arrestment and to reproduce them in the same degree as observed in natural infections. Regarding the histological examination, a common histopathological pattern was repeatedly observed in most of them, though many challenged R fish did not present detectable merogonial or sporogonial stages at certain samplings. This pattern consisted of hypercellularity in the epithelium and lamina propria-submucosa, vacuolation of enterocytes, sometimes showing abnormal nuclei, proliferation and infiltration of granulocytes, proliferation of rodlet cells in the epithelium, and presence of debris. These signs have previously been interpreted as initial signs of *E. nucleophila* infection [23,24], and specific ISH studies have shown that they are often associated with high parasite load and merogonial activity [25]. Therefore, considering that histology is less sensitive than molecular diagnosis to detect the parasitic stages, this histopathological pattern might be used as an indicator of presumptive infection, to obtain further samples for confirmation by molecular methods.

In view of all these results, we can conclude that *E. nucleophila* was successfully transmitted by all the tested infection routes. The anal intubation route seems to be the most successful method, since prevalence reached the maximum value and the infection prevailed at high levels for longer than in other challenges using different routes (oral, effluent and cohabitation). However, the clinical disease signs were not reproduced in the same degree as in naturally infected fish. Certain initial signs of infection were observed, since some R groups showed a significant delay in growth, but these effects were not observed consistently in the follow-up period of all the trials. The results indicate that water temperature, time of exposure, the infective status of D fish and the immunocompetence of the R fish are relevant, possibly more than the challenge route per se. Thus, future studies should aim for longer exposure times at lower temperatures and use overtly infected D fish.

## Figures and Tables

**Table 1:** General information on the four different trials performed for *Enterospora nucleophila* horizontal transmission in gilthead sea bream.

Trial	Challenge	Mean Water Temperature (Range) (°C)	Initial Number of Fish	Samplings (Weeks Post-Challenge)	Prevalence of Infection (%) ^2^
1	O-1	17.2 (10.4–26.0)	15R 10D	8, 20	14.3, 0
	CH-1	16.9 (10.4–25.0)	15R 15D	12, 20	0, 8.3
2	O-2	17.0 (13.4–20.1)	30R 15D	4, 8	70, 55.2
	EF-1	22.4 (13.4–22.9)	31R 31D	4, 10	66.7, 87.1
	EF-2	25.0 (21.5–26.8)	31R 20C 20D	4, 8	80.6, 11.8
3	O-3	19.8 (15.0–25.6)	30R 11C 22D ^1^	4, 12	0, 5.5
	O-3-I	19.8 (15.0–25.6)	40R 20C 22D ^1^	4, 12	10, 0
4	A-1	16.2 (13.7–18.1)	30R 37C 11D	4, 8, 12	70, 100, 100

Four different routes of infection were assayed: oral intubation (O), cohabitation (CH), effluent exposure (EF), and anal intubation (A). I = immunosuppressed fish; D = donor fish; R = recipient fish; C = control non-challenged fish. ^1^ The inoculum for O-3 and O-3-I was prepared using the same 22 donor fish. ^2^ The prevalence values correspond to the sampling times depicted in the previous column.

**Table 2:** Results of trial 2. *Enterospora nucleophila* horizontal transmission in gilthead sea bream was tested by oral intubation (O) and effluent exposure (EF). Fish were diagnosed by non-lethal (NL) or lethal (L) qPCR at two sampling times (S1: 4 weeks post-challenge (wpc) for all challenges; S2: 8 wpc for O-2 and EF2, 10 wpc for EF-1).

Item	O-2	EF-1	EF-2
**Prevalence at S1 (%)**			
NL-qPCR-Blood	0/10 (0%)	28/30 (93.3%)	
NL-qPCR-Intestine	21/30 (70%)	10/30 (66.7%)	25/31 (80.6%)
**Median Ct (range) at S1**			
NL-qPCR-Blood	-	35.8 (34.3–36.9)	
NL-qPCR-Intestine	33.5 (28.7–36.0)	35.8 (34.7–37.9)	36.2 (33.4–37.9)
**Prevalence at S2 (%)**			
NL-qPCR-Blood	4/10 (40%)	3/10 (30%)	
NL-qPCR-Intestine	16/29 (55.2%)	27/31 (87.1%)	
L-qPCR-Intestine (whole)	6/10 (60%)	10/10 (100%)	2/17 (11.8%)
**Median Ct (range) at S2**			
NL-qPCR-Blood	36.0 (34.5–37.8)	36.8 (35.1–37.5)	
NL-qPCR-Intestine	36.7 (31.2–37.6)	35.1 (30.3–37.8)	
L-qPCR-Intestine (whole)	36.3 (35.7–37.3)	33.3 (32.8–34.4)	37.3 (36.7–37.9)
**Biometry at S1**			
Weight (g) C			135 ± 4.71
Weight (g) R	43.0 ± 1.35	48.1 ± 2.03	101.0 ± 3.79 ***
Length (cm) C			17.6 ± 0.18
Length (cm) R	12.6 ± 0.14	13.1 ± 0.17	16.1 ± 0.19 ***
CF C			2.5 ± 0.05
CF R	2.2 ± 0.03	2.1 ± 0.04	2.1 ± 0.14 *
**Biometry at S2**			
Weight (g) C			176.1 ± 5.72 ***
Weight (g) R	44.4 ± 1.49	48.1 ± 2.07	115.9 ± 5.89 ***
Length (cm) C			19.2 ± 0.21 ***
Length (cm) R	12.8 ± 0.15	13.3 ± 0.19	17.0 ± 0.25 ***
CF C			2.5 ± 0.03
CF R	2.1 ± 0.03	2.1 ± 0.12	2.3 ± 0.02 *
**Final Mortality (%)**			
C			1/20 (5%)
R	1/30 (3.3%)	1/31 (3.2%)	4/15 (12.9%)
D	n.a.	17/31 (54.8%)	5/20 (25%)

R = recipient fish; C = control non-challenged fish; D = donor fish; CF = condition factor; n.a. = not available. Lethal qPCR in a homogenate of whole intestine. Asterisks indicate significant differences with the respective non-challenged control group: *p* < 0.05 (*) and *p* < 0.0001 (***).

**Table 3:** Results of trial 3. *Enterospora nucleophila* horizontal transmission in gilthead sea bream by oral intubation of control (O-3) and immunosuppressed fish (O-3-I). Fish were diagnosed by non-lethal (NL) or lethal (L) qPCR at two sampling times (S1: 4 wpc, S2: 12 wpc).

Item	O-3	O-3-I
**Prevalence at S1 (%)**		
NL-qPCR-Intestine	0/10 (0%)	1/10 (10%)
**Median Ct (range) at S1**		
NL-qPCR-Intestine	-	34.5 (34.5)
**Prevalence at S2 (%)**		
L-qPCR-Intestine	1/18 (5.5%)	0/2 (0%)
**Median Ct (range) at S2**		
L-qPCR-Intestine	32.4 (32.4)	-
**Biometry at t0**		
Weight (g) C	9.5 ± 0.46	9.5 ± 0.35
Weight (g) R	10.1 ± 0.34	9.4 ± 0.27
Length (cm) C	7.7 ± 0.10	7.8 ± 0.11
Length (cm) R	8.2 ± 0.17	7.9 ± 0.08
CF C	2.1 ± 0.06	2.0 ± 0.04
CF R	2.1 ± 0.06	2.0 ± 0.02
**Biometry at S1**		
Weight (g) C	n.a.	11.0 ± 0.65
Weight (g) R	10.5 ± 0.38	8.9 ± 0.28 *
Length (cm) C	No data	8.3 ± 0.18
Length (cm) R	8.1 ± 0.09	7.9 ± 0.08 *
CF C	n.a.	1.9 ± 0.06
CF R	2.0 ± 0.05	1.8 ± 0.03
**Biometry at S2**		
Weight (g) C	46.6 ± 2.45	33.6 ± 1.53
Weight (g) R	26.0 ± 1.60***	10.3 ± 1.02 ***
Length (cm) C	12.5 ± 0.15	11.5 ± 0.16
Length (cm) R	10.8 ± 0.23***	8.4 ± 0.10 ***
CF C	2.4 ± 0.06	2.2 ± 0.04
CF R	2.0 ± 0.03***	1.7 ± 0.11 ***
**Final Mortality (%)**		
C	1/11 (9.1%)	4/20 (20%)
R	12/30 (40%)	34/40 (85%)

R = recipient fish; C = control non-challenged fish; n.a. = not available. Asterisks indicate significant differences with the respective non-challenged control groups: *p* < 0.05 (*) and *p* < 0.0001 (***).

**Table 4:** Infection values of *Enterospora nucleophila* in experimentally challenged gilthead sea bream by anal intubation (A-1, Trial 4). Fish were diagnosed by lethal qPCR of intestine at 4-, 8- and 12-weeks post-challenge (wpc). qPCR positive cut-off was Ct < 38). n.a = not available.

	Sampling Time					
Tissue	4 wpc		8 wpc		12 wpc	
	Ct Median (range)	Prevalence (%)	Ct Median (range)	Prevalence (%)	Ct Median (range)	Prevalence (%)
Blood	-	0/10 (0%)	37.2 (37.2)	1/10 (10%)	37.0 (36.0–37.0)	3/10 (30%)
Brain	36.7 (36.7)	1/10 (10%)	-	0/10 (0%)	-	0/10 (0%)
Gallbladder	-	0/10 (0%)	36.0 (36.0)	1/10 (10%)	37.2 (36.5–37.6)	2/10 (20%)
Gills	36.2 (35.4–37.8)	8/10 (80%)	36.3 (36.3)	1/10 (10%)	37.5 (36.5–37.8)	4/10 (40%)
Heart	34.3 (33.3–37.3)	5/10 (50%)	37.0 (36.1–37.8)	4/10 (40%)	37.3 (36.0–37.7)	3/10 (30%)
Head kidney	37.3 (36.6–37.9)	3/10 (30%)	37.1 (37.1)	1/10 (10%)	37.6 (37.6)	1/10 (10%)
Intestine	32.0 (27.5–37.2)	7/10 (70%)	33.5 (26.3–35.7)	10/10 (100%)	30.9 (26.1–37.6)	10/10 (100%)
Liver	33.4 (31.8–37.6)	7/10 (70%)	37.0 (35.7–37.5)	8/10 (80%)	35.4 (33.0–37.7)	2/10 (20%)
Posterior kidney	–	0/10 (0%)	37.5 (37.5)	1/10 (10%)	36.0 (35.0–37.0)	2/10 (20%)
Spleen	34.6 (33.1–35.5)	2/10 (20%)	37.0 (36.7–37.3)	2/10 (20%)	36.0 (36.0)	1/10 (10%)
Stomach	34.2 (26.2–37.9)	8/10 (80%)	34.4 (31.7–37.4)	6/10 (60%)	36.4 (35.5–37.3)	2/10 (20%)
Swim bladder	n.a.	n.a.	35.7 (34.8–37.9)	4/10 (40%)	37.5 (36.5–37.6)	3/10 (30%)

## Data Availability

All the data is available in the article and supplementary file.

## Supplementary Materials

The following are available online at https://www.mdpi.com/2076-2615/11/2/362/s1, Table S1: *Enterospora nucleophila* infection status in the intestine of gilthead sea bream used as donors in the different infection trials.
